# ‘If I am Reminded of my Trauma, I will …’: Assessing Threat Expectancies for Being Confronted with Trauma Reminders

**DOI:** 10.1007/s10608-025-10582-5

**Published:** 2025-02-20

**Authors:** Marike Jolien Kooistra, Agnes van Minnen, Danielle Oprel, Maartje Schoorl, Willem van der Does, Rianne de Kleine

**Affiliations:** 1 Department of Clinical Psychology, Leiden University, Leiden, The Netherlands; 2 PsyQ, Parnassia Groep, The Hague, The Netherlands; 3 Leiden University Treatment Center (LUBEC), Leiden, The Netherlands; 4 Behavioural Science Institute, Radboud University Nijmegen, Nijmegen, The Netherlands

**Keywords:** Posttraumatic stress disorder, Threat appraisal, Posttraumatic cognitions, Assessment, Validation

## Abstract

**Purpose:**

Dysfunctional threat appraisal plays a key role in both the development and treatment of PTSD. It is unclear how these appraisals can best be measured. This study aimed to explore the specific negative outcome predictions held by patients with PTSD and to develop and validate the Threat Appraisal in PTSD Scale (TAPS).

**Methods:**

We used data from a non-clinical (*N* = 309) and clinical sample (*N* = 125) to assess the psychometric properties of the TAPS.

**Results:**

The TAPS had excellent internal consistency and test-retest reliability, and convergent and discriminative validity were adequate. The TAPS showed to be sensitive to change following treatment. The TAPS demonstrated incremental validity beyond general cognitions in predicting PTSD symptoms in the combined sample, but not in the patient sample. An exploratory factor analysis suggested three factors: ‘losing control’, ‘externalizing reactions’, and ‘physical reactions’, and patients seemed most concerned about outcomes related to ‘losing control’.

**Conclusions:**

These findings imply that the TAPS could be clinically beneficial, enabling patients and therapists to recognize dysfunctional expectancies and tailor therapeutic interventions accordingly.

**Supplementary Information:**

The online version contains supplementary material available at 10.1007/s10608-025-10582-5.

## Introduction

People who suffer from posttraumatic stress disorder (PTSD) tend to hold negative beliefs about themselves, others, and the world. In different theoretical models of PTSD, negative trauma-related cognitions about the trauma and its sequala have been suggested to be central in PTSD symptom development and maintenance (Ehlers & Clark, 2000; Rauch & Foa, 2006; Resick & Schnicke, 1992). Indeed, many empirical studies have underscored the centrality of negative cognitions and its relationship with the onset, maintenance, and recovery from PTSD (Brown et al., 2019; Gómez de La Cuesta et al., 2019). With regard to PTSD treatment, changes in negative cognitions predict subsequent changes in other PTSD symptoms, and changing negative cognitions have therefore been proposed as one of the mechanisms of change during treatment (Alpert et al., 2023; Cooper et al., 2017).

To underscore its importance, persistent negative alterations in cognitions were added to the diagnostic criteria of PTSD in the DSM-5 (American Psychiatric Association, 2013). Expectancies are considered a subgroup of cognition and include specific predictions about the likelihood of future events or experiences (Herzog et al., 2023; Rief et al., 2015). Dysfunctional expectancies are presumed to be closely related to more general negative beliefs. For instance, someone may hold the negative belief that the world is dangerous and may therefore wrongfully expect to be attacked when going out. Negative expectancies are theorized to be overestimated in both likelihood and cost by individuals with PTSD (Ehlers & Clark, 2000; Rauch & Foa, 2006). Moreover, experimental psychopathology studies have shown that negative threat expectancies are related to the development and severity of PTSD symptoms (Engelhard et al., 2009; Herzog et al., 2022; Kimble et al., 2018). For instance, negative expectancies about the intensity and uncontrollability of intrusions following a trauma-film paradigm were predictive of PTSD intrusion symptom development one week later (Herzog et al., 2022). As expectancies are generally formulated in ‘if-then’ statements, they are suitable targets for therapeutic interventions such as behavioral experiments and exposure exercises.

Given that elevated threat expectancies appear to be an important feature of PTSD and a treatment target, it would be useful to have a measure that specifically gauges these cognitions. Several instruments that measure (trauma-related) cognitions already exist, such as the Posttraumatic Cognitions Inventory (PTCI; Foa et al., 1999), the Posttraumatic Growth Inventory (PTGI; Tedeschi & Calhoun, 1996), the Posttraumatic Maladaptive Beliefs Scale (PMBS; Vogt et al., 2012), the Dissociation-Related Beliefs about Memory Questionnaire (DBMQ; Huntjens et al., 2023) and the Metacognitions Questionnaire (MCQ-30; Wells & Cartwright-Hatton, 2004). However, these questionnaires seem to primarily measure general or meta cognitions rather than specific expected negative outcomes. The PTCI, the most commonly used instrument to assess negative trauma-related cognitions, includes only a few future-oriented items with just one framed as an if-then statement (“If I think about the event, I will not be able to handle it”). Specific predictions about negative outcomes in relation to future trauma-related events or experiences are therefore barely covered.

For social anxiety disorder, a measure does exist that assesses expected negative outcomes in social events (the Appraisal of Social Concerns scale; ASC; Schultz et al., 2006; Telch et al., 2004). More specifically, this 20-item questionnaire measures the concern for concrete negative outcomes (e.g., ‘people laughing at you’ and ‘appearing weird’) in future challenging social situations. This measure proved valid and has been used to tailor treatment sessions and evaluate treatment effects (Krafft et al., 2020; Laposa & Rector, 2023; Winkler et al., 2022). Based on this instrument, we developed a scale that assesses threat expectancies for trauma-related events or experiences for those suffering from PTSD. Recently, a similar measure has been developed, the Posttraumatic Expectations Scale (Herzog et al., 2023), which covers a broad range of PTSD and treatment related expectancies. In a sample of 70 treatment-seeking patients suffering from PTSD, the authors found that expectancies explained additional variance in predicting PTSD symptom severity over the effect of more general negative trauma-related cognitions (as assessed with the PTCI). The full version of the PTES contains 81 items and is thereby quite lengthy. Furthermore, not all subscales of the measure appeared to be reliable. The authors also developed a short version (13 items), but this version only has one item that assesses an expectation related to confrontation with a trauma-reminder (‘When I am reminded of the traumatic event, I will feel that the world around me is not real’). Our measure specifically focuses on concerns about concrete and testable negative outcomes in response to trauma reminders. The assessment of negative expectations related to confrontation with trauma-reminders may have great clinical utility, as (imaginal) exposure to trauma-reminders is a common and critical element of empirically supported psychotherapeutic treatments for PTSD (Schnyder et al., 2015). Patients often struggle to identify concrete negative expectancies, and having a valid instrument may increase awareness while helping therapists design interventions that target dysfunctional predictions and optimize treatment outcomes.

The aim of the current study is to advance the assessment of commonly perceived threats in response to confrontation with trauma-related stimuli or situations in patients with PTSD. We created a 24-item self-report measure called the Threat Appraisal in PTSD Scale (TAPS). Individuals are asked to rate their level of concern about anticipated specific negative outcomes of confrontation with trauma reminders (e.g., ‘not being able to talk’ or ‘fainting’). Using a non-clinical and a patient sample, we report on the development of the measure and its psychometric properties: internal consistency, factor structure, discriminative, convergent and incremental validity, and sensitivity to change over the course of treatment.

## Methods

### Scale and Item Development

The instructions and scoring of the TAPS were based on the ASC (Schultz et al., 2006; Telch et al., 2004). Multiple sources were used to create items for the current measure. First, items were generated by reviewing data from the IMPACT study, a large randomized controlled trial on the effectiveness of three variants of exposure therapy (Oprel et al., 2018). In the IMPACT study, 149 patients reported idiosyncratic concrete outcomes they feared when confronted with a trauma-reminder (in total, this dataset contained 1385 idiosyncratic feared outcomes). These outcomes were reviewed and clustered, and formed the basis for the TAPS. We also examined similar, previously developed, scales (i.e., scales that assess cognitions in the context of PTSD and anxiety disorders). Finally, we let three international experts in the field of PTSD and exposure therapy review all generated items, which led to the addition and reformulation of several items. We ended up with 24 items for the questionnaire. Similar to the ASC, we chose to ask participants to rate their degree of concern about a negative anticipated outcome, aiming to capture its perceived likelihood and cost, whilst keeping the measure concise and easy to administer. Participants are asked to rate their level of concern for a negative outcome when confronted with a trauma reminder, ranging from 0 (‘not at all concerned’) to 100 (‘extremely concerned’), where a score of 50 represents moderate concern. The TAPS total score is calculated by taking the individual’s mean on all items.

### Participants

A nonclinical sample (*N* = 309) was recruited via university campus advertisements. Individuals from this nonclinical sample were excluded if they had not experienced a traumatic or severely stressful event in the past, as defined by the Life Events Checklist for the DSM-5 (LEC-5). Furthermore, potential participants were excluded if they reported a current diagnosis of a mental disorder and/or were receiving professional help for a mental disorder or psychological problems at the time of the study. A clinical sample of adult patients with PTSD (*N* = 125) was recruited via two out-patient clinics specializing in the treatment of PTSD. Individuals from this clinical sample were included if they satisfied DSM-5 criteria for PTSD assessed by clinical interview (SCID-S or CAPS-5). Patients were excluded if they had insufficient ability to speak and read Dutch and/or if their estimated IQ was below 70. Data from the non-clinical sample was collected from January 2021 to April 2022. Data from the patient sample was collected from November 2020 to September 2024.

### Measures

#### Negative Life Events

The Life Events Checklist for the DSM-5 (LEC-5; Weathers et al., 2013) was used to identify the traumatic events participants had experienced. The self-report questionnaire contains 16 items on distressing events where participants can respond with ‘happened to me’, ‘witnessed it’, ‘learned about it’, ‘part of my job’, ‘not sure’, or ‘does not apply’. One item (item 17) is open-ended where participants can identify a severely stressful event that was not listed before.

#### Childhood Trauma

The short version of the Childhood Trauma Questionnaire (CTQ-SF; Bernstein et al., 2003) was used to assess the extent of childhood trauma in the samples. The CTQ-SF is a 28-item self-report questionnaire. Each item is rated on a 5-point Likert scale, ranging from ‘never true’ (1) to ‘very often true’ (5). The total score ranges from 25 to 125, where higher scores reflect more childhood trauma. The measure contains five subscales: emotional abuse, physical abuse, sexual abuse, emotional neglect and physical neglect.

#### PTSD Symptomatology

The PTSD checklist for DSM-5 (PCL-5; Blevins et al., 2015; Hoeboer et al., 2024) was used to assess PTSD symptoms. The PCL-5 is a 20-item self-report questionnaire. Each item is rated on a 4-point Likert scale, ranging from ‘not at all’ (0) to ‘extremely’ (4). The total score is calculated by summing all items and ranges from 0 to 80, where higher scores reflect higher symptom severity. The PCL-5 has good psychometric properties, with a high internal consistency (including in the present non-clinical and patient samples, Cronbach’s α = 0.91 and 0.89 respectively) and good validity (Hoeboer et al., 2024).

#### Posttraumatic Cognitions

The Posttraumatic Cognitions Inventory (PTCI; Foa et al., 1999; van Emmerik et al., 2006) was used to assess trauma-related cognitions. The PTCI is a 33-item self-report questionnaire. Each item is rated on a 7-point Likert scale, ranging from ‘totally disagree’ (1) to ‘totally agree’ (7). The total score is calculated by summing all items and ranges from 33 to 231, where higher scores reflect more trauma-related cognitions. The PTCI total score has adequate psychometric properties. Internal consistency is high (including in the present non-clinical and patient samples, Cronbach’s α = 0.94 and 0.95 respectively).

#### Intolerance of Uncertainty

The Intolerance of Uncertainty Scale-Short Form (IUS-12; Carleton et al., 2007; Helsen et al., 2013) was used to assess the tendency to find a potential negative event unacceptable, regardless of how likely it is to happen. The IUS-12 is a 12-item self-report questionnaire where each item is rated on a 5-point Likert scale, ranging from ‘not at all characteristic of me’ (1) to ‘entirely characteristic of me’ (5). The total score ranges from 5 to 60, where higher scores reflect more intolerance of uncertainty. The psychometric properties have been shown to be strong (Boelen et al., 2010; Carleton et al., 2007), with high internal consistency (including in the present non-clinical and patient samples, Cronbach’s α = 0.90 and 0.87 respectively).

### Procedures

The study was approved by the Leiden University Psychology Ethics Committee (#2022-11-23-R.A.de Kleine-V1-4357). All participants provided informed consent before participating in the study. In the non-clinical sample, eligible and interested participants received a link to online questionnaires. Before starting the questionnaires, participants were asked whether they had experienced a very stressful or traumatic event (yes/no). Only those who answered ‘yes’ were redirected to complete the questionnaires. Nine participants did not finish the entire set of questionnaires, leading to missing data on questionnaires that followed the TAPS. The total sample (*N* = 309) completed the TAPS, 307 participants completed the PCL-5, 304 completed the PTCI, 303 completed the CTQ, and 300 completed the IUS-12. A number of participants in the non-clinical sample (*n* = 158, 51.1%) was asked to fill out the TAPS again one week later, in order to assess test-retest reliability. The patient sample had to fill out the questionnaires within the first two months of treatment. Questionnaires were completed online, but patients who were unable to do so were given the option to complete them on paper. Again, not all participants completed the full set of questionnaires. The total sample (*N* = 125) completed the TAPS, of which 123 participants completed the PCL-5, 124 completed the PTCI, 123 completed the CTQ, and 101 completed the IUS-12. A number of participants in the patient sample (*n* = 80, 64.0%) completed the questionnaires as part of their participation in other treatment studies (Kooistra et al., 2025; Kooistra et al., *in preparation*).

Additionally, to test sensitivity to change, a number of participants in the patient sample (*n* = 41, 32.8%) was asked to fill out the questionnaires pre and post PTSD treatment. Participants all received intensified Prolonged Exposure therapy for PTSD (iPE; Foa et al., 2019; Oprel et al., 2021), which was delivered in 12 to 14 face-to-face sessions of 90-minutes of PE, with 3 sessions per week for 4 weeks. Treatment included psycho-education, imaginal exposure, and exposure in vivo. Between sessions, patients were instructed to do homework assignments (e.g., listening to audiotaped exposure sessions and exposure in vivo exercises). Participants completed the PCL-5, PTCI and TAPS for the second time three months after starting treatment.

### Statistical Analyses

We provide descriptive information on the TAPS items in the non-clinical and patient sample, such as item mean and standard deviation. We also assessed which items were, on average, rated as most concerning by making a ranked list of items (from most to least concerning). To examine the underlying factors in the TAPS, we conducted an exploratory factor analysis (EFA) using Principal Axis Factoring (PAF) as extraction method on the combined sample (non-clinical and patient samples). PAF was chosen as the TAPS items were not normally distributed. When developing the scale, we did not have a priori hypotheses about its potential underlying factors. As the TAPS is primarily intended for clinical populations, analyzing the patient sample alone would have been ideal, but this sample was relatively small. We used oblimin rotations as the factors were expected to correlate. Eigenvalues, the scree method, factor loadings and fit statistics were assessed to derive the underlying factor structure of the scale. Discriminative validity was assessed by testing whether the TAPS was significantly higher in the patient sample through an independent sample t-test. Internal consistency was assessed in the combined and patient sample through Cronbach’s α and McDonald’s ω, with a value of ≥ 0.7 indicating sufficient reliability. The ‘Cronbach’s α if Item Deleted’ was assessed to identify items that lower the overall internal consistency of the scale. The test-retest reliability of the TAPS was assessed using the subsample of the non-clinical individuals by calculating the Spearman correlation between the first time it was administered and the second time it was administered (a week later). Spearman was chosen as the TAPS was not normally distributed. Convergent validity was examined by calculating Spearman correlations between the TAPS, the PCL-5, the PTCI, and the IUS-12. Incremental validity of the TAPS was assessed via a multiple hierarchical regression analysis, with the PCL-5 as dependent variable and the PTCI and the TAPS as independent variables (added in separate steps). Finally, to assess whether the TAPS was sensitive to change due to Prolonged Exposure therapy, we conducted a multilevel analysis with random intercept, where Time (pre- and posttreatment) was entered as the independent variable and TAPS as dependent variable. We explored whether potential TAPS subscales were also sensitive to treatment. We carried out the same analysis for the PCL-5 to gauge whether treatment was effective. Finally, we assessed the relationship between change in TAPS (TAPS_pre_ – TAPS_post_) and change in PCL-5 (PCL-5_pre_ – PCL-5_post_) by calculating a Pearson correlation. Analyses were carried out in SPSS version 29, except for the multilevel analyses. Multilevel analyses were tested in R (Version 4.0.1) with maximum likelihood estimation using the lme4 package (v1.1-28; Bates et al., 2015). Alpha level for all analyses was set at 0.05. This study was preregistered at OSF (https://osf.io/av8e9/?view_only=6891ee2e0cff4256a8212c0f64850dfc).

## Results

### Sample Characteristics

The sample characteristics are shown in Table 1. Between the groups, there were significant differences in age (the non-clinical sample was younger), *t*(432) = -11.67, *p* <.001, but not in gender, χ²(2, *N* = 434) = 5.27, *p* =.072. The patient group reported greater severity of childhood trauma (CTQ; *t*(424) = -18.08, *p* <.001), more PTSD symptoms (PCL-5, *t*(428) = -25.34, *p* <.001), higher levels of negative trauma-related cognitions (PTCI, *t*(426) = -20.10, *p* <.001), and higher intolerance of uncertainty (IUS-12, *t*(399) = -9.98, *p* <.001).

As assessed with the LEC-5, in the nonclinical sample, the most frequently endorsed concrete negative life event that was directly experienced or witnessed was an unwanted or uncomfortable sexual experience (*n* = 123, 39.8%), followed by physical assault (*n* = 113, 36.6%), and a life-threatening illness or injury (*n* = 115, 37.2%). In the patient sample, the most experienced or witnessed was physical assault (*n* = 116, 92.8%), followed by an unwanted or uncomfortable sexual experience (*n* = 97, 77.6%), and sexual assault (*n* = 93, 74.4%). Additionally, 47.2% (*n* = 146) of the non-clinical sample and 80.8% (*n* = 101) of the patient sample reported to have experienced or witnessed another negative life event, such as being bullied or a divorce. Patients reported to have experienced on average 7.3 types of potentially traumatic events (*SD* = 2.8), and non-clinical participants on average 3.2 (*SD* = 1.9).

**Table 1:** Baseline characteristics of participants

	Non-clinical sample (*N* = 309)	Patient sample (*N* = 125)
Age in years, mean (SD)	23.8 (9.8)	36.9 (12.2)
Gender, *n* (%)		
Male	49 (15.9)	31 (24.8)
Female	259 (83.8)	93 (74.4)
Non-binary	1 (0.3)	1 (0.8)
Number of negative LEs	3.2 (1.9)	7.3 (2.8)
PCL-5, mean (SD)	19.8 (14.1)	55.7 (11.0)
PTCI, mean (SD)	80.7 (32.0)	151.5 (35.4)
CTQ	37.3 (13.2)	69.7 (23.3)
Emotional abuse	8.4 (4.4)	16.7 (6.3)
Physical abuse	6.1 (3.0)	12.5 (6.4)
Sexual abuse	6.0 (2.7)	12.3 (6.6)
Emotional neglect	9.8 (4.2)	17.5 (5.3)
Physical neglect	7.0 (2.8)	11.0 (4.5)
IUS-12	34.1 (9.4)	44.7 (8.7)

Note SD = standard deviation; LEs = life events; PCL-5 = PTSD Checklist for DSM-5; PTCI = Posttraumatic Cognitions Inventory; CTQ = Childhood Trauma Questionnaire; IUS-12 = Intolerance of uncertainty scale, short form

### TAPS Item Analysis

Per TAPS item, the mean score and standard deviation are shown in Table 2 for both samples. In the non-clinical sample, the mean rate of concern per item ranged from 4.1 (item 16 ‘Wetting or soiling my pants’) to 32.6 (item 5 ‘Becoming a victim again/being in danger’). In the patient sample, the mean rate of concern per item ranged from 7.3 (item 16 ‘Wetting or soiling my pants’) to 65.8 (item 12 ‘Unable to think, having a black out’). The ranking of items, based on their mean scores (with the highest mean score assigned the highest rank), was consistent across samples, with the same items appearing in the top five positions. Clinically, it is especially relevant to identify anticipated negative outcomes for which patients have a high level of concern (Craske et al., 2022). Therefore, we counted how many concerns about negative outcomes were rated 60 or higher (see also, Craske et al., 2022). In the non-clinical sample, participants had on average 2.3 items above 60 (*SD* = 3.7; range: 0–22) and, 135 participants (43.7%) had no concern rated over 60. In the patient sample, participants had on average 8.1 items above 60 (*SD* = 5.0; range: 0–23), and seven participants (5.6%) had no concern rated over 60.

**Table 2:** Overview of TAPS items, including their mean, ordered by rank in the patient sample

		Non-clinical sample			Patient sample		
Item		M (SD)	Rank	> 60, *n* (%)	M (SD)	Rank	> 60, *n* (%)
12	Unable to think (having a blackout)	27.0 (30.1)	3	57 (18.4)	65.8 (31.6)	1	88 (70.4)
5	Becoming a victim again/being in danger	32.5 (32.7)	1	88 (28.5)	60.3 (35.6)	2	76 (60.8)
24	Unable to function	21.9 (29.6)	5	50 (16.2)	58.5 (33.1)	3	71 (56.8)
14	Unable to feel anything	22.1 (29.8)	4	50 (16.2)	57.3 (34.8)	4	72 (57.6)
19	Unable to stop crying	30.0 (31.6)	2	70 (22.7)	52.8 (37.1)	5	64 (51.2)
21	Walking away or running away	16.4 (23.8)	7	21 (6.8)	49.5 (36.5)	6	60 (48.0)
11	Unable to talk	18.4 (27.1)	6	40 (12.9)	47.6 (35.2)	7	55 (44.0)
7	Unable to move	16.1 (27.2)	9	34 (11.0)	42.7 (37.2)	8	51 (40.8)
15	Hurting myself	10.7 (22.5)	15	24 (7.8)	41.1 (36.7)	9	49 (39.2)
13	Swearing or cursing	16.4 (24.9)	7	27 (8.7)	41.0 (37.6)	10	50 (40.0)
9	Not knowing where I am	8.7 (19.9)	17	17 (5.5)	38.8 (34.4)	11	44 (35.2)
1	Screaming	16.0 (23.0)	10	30 (9.7)	34.2 (35.3)	12	38 (30.4)
17	Collapsing	13.5 (24.6)	12	31 (10.0)	31.5 (32.6)	13	33 (26.4)
20	Speaking gibberish	8.0 (17.2)	20	13 (4.2)	31.4 (34.4)	14	34 (27.2)
8	Fainting	13.8 (24.4)	11	24 (7.8)	30.8 (30.5)	15	30 (24.0)
23	Moving uncontrollably	7.4 (16.8)	21	12 (3.9)	29.9 (33.5)	16	31 (24.8)
3	Vomiting	13.1 (23.7)	13	25 (8.1)	28.5 (30.7)	17	27 (21.6)
2	Throwing things	10.9 (18.7)	14	14 (4.5)	28.5 (32.4)	18	29 (23.2)
10	Hitting or kicking	8.6 (18.9)	18	14 (4.5)	28.4 (34.4)	19	32 (25.6)
6	Choking	6.2 (15.3)	22	9 (2.9)	25.3 (33.2)	20	24 (19.2)
18	Dying	9.0 (21.9)	16	21 (6.8)	20.1 (32.6)	21	23 (18.4)
22	Hurting someone else	5.1 (15.4)	23	10 (3.2)	19.1 (29.4)	22	15 (12.0)
4	Having a heart attack	8.4 (19.9)	19	17 (5.5)	16.9 (25.9)	23	14 (11.2)
16	Wetting or soiling my pants	4.1 (13.5)	24	6 (1.9)	7.3 (18.4)	24	5 (4.0)

*Notes M* = mean; *SD* = standard deviation; Rank = relative standing of the item based on highest mean; >60 = number of participants who rated that item as higher than 60

### Exploratory Factor Analysis

All items were entered in an EFA with oblimin rotation. Kaiser’s criterion (i.e., eigenvalues > 1) favoured a four-factor solution, while the Scree method favoured a two-factor solution. We therefore explored a two, three and four factor structure. The three-factor structure proved to be the best fit, based on the assessment of cross-loading items, non-loading items, communalities of items and interpretability (see supplementary material for the other factor solutions). The three-factor model explained 50.4% of the variance. We used a cutoff of 0.40 for factor loadings to determine the items that meaningfully contributed to the factor in the analysis. Table 3 provides an overview of the factors, and the factor loading and communality of each item. We labelled the first factor, consisting of 11 items, ‘losing control’, the second factor, 5 items, ‘externalizing reactions’ and the third factor, 5 items, ‘physical reactions’. Three items did not appear to load on any factor (item 23 ‘Moving uncontrollably’, item 17 ‘Collapsing’, and item 3 ‘Vomiting’), and were therefore removed from the final scale. The correlation between the factors were moderate, ranging from *r* =.38 to *r* =.55.

The final scale thus consisted of 21 items. The patient sample had a significantly higher total score (*M* = 38.0, *SD* = 18.6) than the non-clinical sample (*M* = 14.8, *SD* = 14.0), *t*(432) = -14.17, *p* <.001. The subscales ‘losing control’ (*M* = 49.6, *SD* = 22.9 vs. *M* = 19.2, *SD* = 17.7), ‘externalizing reactions’ (*M* = 30.3, *SD* = 26.1 vs. *M* = 11.4, *SD* = 15.5), and ‘physical reactions’ (*M* = 20.1, *SD* = 19.9 vs. *M* = 8.3, *SD* = 14.7) were also significantly higher in the patient sample, *t*(432) = -14.81, -9.29, and − 6.78, respectively, all *p* <.001.

### Reliability

The internal consistency of the TAPS was excellent in both the combined sample (Cronbach’s α = 0.93, McDonald’s ω = 0.94) and the patient sample (Cronbach’s α = 0.89, McDonald’s ω = 0.89). Internal consistency would not improve by deleting any item, suggesting that all items contribute to internal consistency. Internal consistency was also good for all subscales; ‘losing control’ (combined sample: Cronbach’s α = 0.91, McDonald’s ω = 0.91; patient sample: Cronbach’s α = 0.86, McDonald’s ω = 0.86), ‘externalizing reactions’ (combined sample: Cronbach’s α = 0.85, McDonald’s ω = 0.85; patient sample: Cronbach’s α = 0.83, McDonald’s ω = 0.83), and ‘physical reactions’ (combined sample: Cronbach’s α =.80, McDonald’s ω =.81; patient sample: Cronbach’s α =.73, McDonald’s ω =.74).

We also assessed the temporal stability of the TAPS in the non-clinical sample. Out of the 158 participants in the non-clinical sample who were asked to fill out the TAPS questionnaire one to two weeks later, 127 participants did so. The Spearman showed a strong positive correlation between the TAPS at both timepoints, *r* =.77, *p* <.001, indicating good test-retest reliability (i.e., temporal stability).

**Table 3:** 3-factor pattern loadings and communalities

		Factor 1	Factor 2	Factor 3	
Items		Losing control	Externalizing reactions	Physical reactions	h^2^
12.	Unable to think (having a blackout)	**0.913**	− 0.139	− 0.008	**0.71**
11.	Unable to talk	**0.816**	0.007	− 0.068	**0.62**
24.	Unable to function	**0.781**	− 0.037	0.083	**0.65**
14.	Unable to feel anything	**0.702**	0.013	0.017	**0.52**
7.	Unable to move	**0.668**	− 0.015	0.066	**0.49**
9.	Not knowing where I am	**0.623**	0.025	0.196	**0.58**
5.	Becoming a victim again/being in danger	**0.569**	0.044	0.034	0.38
20.	Speaking gibberish	**0.559**	0.116	0.078	**0.46**
19.	Unable to stop crying	**0.531**	0.128	− 0.089	0.32
21.	Walking away or running away	**0.493**	0.294	− 0.117	**0.42**
15.	Hurting myself	**0.486**	0.211	0.094	**0.46**
17.	Collapsing	0.388	0.082	0.387	**0.52**
23.	Moving uncontrollably	0.387	0.106	0.274	**0.42**
2.	Throwing things	− 0.013	**0.742**	0.129	**0.63**
1.	Screaming	0.021	**0.740**	− 0.024	**0.55**
10.	Hitting or kicking	0.079	**0.724**	0.057	**0.63**
13.	Swearing or cursing	0.158	**0.654**	− 0.130	**0.50**
22.	Hurting someone else	− 0.085	**0.581**	0.322	**0.50**
4.	Having a heart attack	− 0.059	0.045	**0.821**	**0.65**
18.	Dying	0.020	0.031	**0.676**	**0.49**
16.	Wetting or soiling my pants	0.019	0.099	**0.530**	0.34
8.	Fainting	0.365	− 0.109	**0.481**	**0.48**
6.	Choking	0.212	0.162	**0.450**	**0.47**
3.	Vomiting	0.272	− 0.006	0.378	0.32
Eigenvalue		8.40	5.71	5.57	
(Additional) % of variance		39.44	5.78	5.15	

*Notes h*^*2*^ = communalities; Factor loadings in bold indicate that the item loads on a factor (with a cutoff of 0.40)

### Convergent and Incremental Validity

To assess convergent validity, we calculated Spearman correlations between the TAPS and measures that it should theoretically be related to, see Table 4. In the combined sample, the TAPS and its subscales were significantly and positively correlated with all other measures (PCL-5, PTCI, and IUS-12). In the patient sample, the TAPS and its subscales ‘losing control’ and ‘physical reactions’ significantly and positively correlated with the other measures. Externalizing reactions did not correlate significantly with the other measures.

**Table 4:** Correlations of TAPS and theoretically related measures

Combined sample	1.	2.	3.	4.	5.	6.	7.
1. TAPS	-	-	-	-	-	-	-
2. TAPS-LC	0.96***	-	-	-	-	-	-
3. TAPS-ER	0.72***	0.58***	-	-	-	-	-
4. TAPS-PR	0.68***	0.59***	0.45***	-	-	-	-
5. PCL-5	0.70***	0.70***	0.43***	0.44***	-	-	-
6. PTCI	0.67***	0.68***	0.43***	0.41***	0.82***	-	-
7. IUS-12	0.52***	0.53***	0.36***	0.30***	0.59***	0.63***	-
Patient sample							
1. TAPS	-	-	-	-	-	-	-
2. TAPS-LC	0.92***	-	-	-	-	-	-
3. TAPS-ER	0.65***	0.38***	-	-	-	-	-
4. TAPS-PR	0.67***	0.51***	0.29**	-	-	-	-
5. PCL-5	0.32***	0.32***	0.09	0.19*	-	-	-
6. PTCI	0.38***	0.40***	0.17	0.20*	0.57***	-	-
7. IUS-12	0.36***	0.38***	0.14	0.22*	0.59***	0.63***	-

*Notes* TAPS = Threat Appraisal in PTSD Scale; TAPS-LC = Losing control TAPS subscale; TAPS-ER = Externalizing reactions TAPS subscale; TAPS-PR = Physical reactions TAPS subscale; PCL-5 = PTSD Checklist for DSM-5; PTCI = Posttraumatic Cognitions Inventory; IUS-12 = Intolerance of uncertainty scale, short form; *p <.05; ** = *p* <.01; ***p <.001

In the combined sample, the hierarchical multiple regression analysis showed that the TAPS provided additional predictive power beyond the PTCI (i.e., incremental validity). In Block 1, PTCI was a significant predictor, R^2^ = 0.70, *F*(1, 425) = 990.87, *p* <.001. Adding TAPS in Block 2 resulted in a significant increase in explained variance, ΔR^2^ = 0.03, *F*(1, 424) = 46.86, *p* <.001, with the final model being significant, R^2^ = 0.73, *F*(2, 424) = 572.33, *p* <.001. Both PTCI (β = 0.31, *p* <.001) and TAPS (β = 0.27, *p* <.001) significantly predicted PCL-5 scores. This was not true for the patient sample, where in Block 1, R^2^ = 0.36, *F*(1, 121) = 67.25, *p* <.001, PTCI was a significant predictor (β = 0.19, *p* <.001). Adding TAPS in Block 2 resulted in no significant increase in explained variance, ΔR^2^ = 0.01, *F*(1, 120) = 1.48, *p* =.227.

### Sensitivity to Treatment

Out of the 41 participants in the patient sample who were asked to fill out the TAPS after treatment, 32 participants did so. In this sample, the PCL-5 significantly decreased from pre-treatment (*M* = 56.5; *SD* = 10.2) to post-treatment (*M* = 28.3; *SD* = 18.5), *b* = − 28.37, *SE* = 1.16, *t* = − 12.42, *p* <.001, showing that the treatment was effective in reducing PTSD symptoms. The TAPS total score also significantly decreased from pre-treatment (*M* = 37.5; *SD* = 14.4) to post-treatment (*M* = 17.9; *SD* = 18.4), *b* = − 19.80, *SE* = 2.81, *t* = − 7.04, *p* <.001, indicating that it is sensitive to treatment-related changes. Moreover, all subscales (losing control, externalizing reactions, and physical reactions) significantly decreased from pre to post-treatment, *b* = − 26.51, *SE* = 3.37, *t* = − 7.87, *p* <.001, *b* = − 13.87, *SE* = 3.63, *t* = − 3.82, *p* <.001, *b* = − 13.89, *SE* = 3.64, *t* = − 3.81, *p* =.001, respectively. Change in the TAPS (i.e., pre-treatment minus post-treatment) was positively, strongly and significantly related with change in PTSD symptoms (i.e., pre-treatment minus post-treatment), *r* =.58, *p* <.001.

## Discussion

The current study presents the initial reliability and validity of a newly developed measure, called TAPS (threat appraisal for PTSD scale), using combined data from a non-clinical and treatment seeking patient sample. Testable and concrete dysfunctional expectancies are considered an important subcategory of negative cognitions, as they can be directly targeted in psychological interventions (e.g., ‘if I recount the traumatic event, I will be unable to stop crying’). Negative cognitions are commonly more generally assessed as beliefs about the self, others and the world (e.g., ‘I am weak’ and ‘The world is a dangerous place’), as in the DSM-5 and the PTCI (American Psychiatric Association, 2013; Foa et al., 1999). We developed the TAPS to capture concrete and testable trauma-related expectancies. The psychometric properties of the TAPS indicate it is a valuable addition to the field. On average, patients recognized to have high concerns for multiple negative outcomes, with considerable variation between participants (e.g., ranging from high concerns for zero items to as many as 23). An exploratory factor analysis reduced the scale from 24 to 21 items with three factors. The TAPS was internally consistent, temporally stable, and correlated to theoretically related constructs. The weak to moderate correlation with the PTCI suggest that the TAPS aligns with this established measure while also potentially capturing unique aspects of trauma-related cognition, reflecting its refined scope. The TAPS was able to distinguish between patients and controls and was sensitive to treatment. In the combined sample, the TAPS also demonstrated incremental validity beyond more general cognitions (PTCI) in predicting PTSD symptoms, although this was not true in the patient sample only.

The first factor of the factor analysis was labeled ‘losing control’. The idea that one is losing mental control is theoretically presumed to maintain a sense of current threat in those suffering from PTSD (Ehlers & Clark, 2000), and trauma-focused treatments, such as Prolonged Exposure, target the erroneous beliefs of ‘loss of control’ and ‘going crazy’ (Foa et al., 2019). Our data shows more concretely what this losing control may look like. Interestingly, a majority of patients (> 50%) rated items relating to dissociative symptoms as highly concerning (e.g., having a black out or being unable to talk or feel anything). This aligns with findings that individuals diagnosed with PTSD and dissociative disorders often hold meta-memory beliefs, perceiving that retrieving and processing traumatic memories may result in negative consequences (e.g., ‘I believe that if I would allow myself to remember, my memories would overwhelm me’; Huntjens et al., 2023). Interestingly, the only item about trauma-reminder confrontation that was retained in the short version of the PTES was also related to dissociation (‘When I am reminded of the traumatic event, I will feel that the world around me is not real’; Herzog et al., 2023), further emphasizing the importance of negative expectancies associated with dissociative responses to trauma reminders. With the TAPS, we present a list that more thoroughly captures such expectations. This factor, ‘losing control’, was most strongly related to PTSD symptoms and general posttraumatic cognitions, in both the combined (strong correlations) and patient sample (moderate correlations).

The second factor comprised items referring to concerns about externalizing reactions, and was labeled as such. Externalizing reactions are included in the arousal symptom cluster of PTSD (i.e., ‘irritable behavior and angry outburst’), and anger difficulties seem more pronounced in PTSD compared to other anxiety-based disorders (American Psychiatric Association, 2013; Olatunji et al., 2010). Surprisingly, concerns about externalizing reactions did not relate to more severe PTSD symptoms in the patient sample, although it was significantly higher in this sample compared to healthy controls. Previous research has suggested that the link between anger and PTSD is more pronounced in men (Taft et al., 2017). It would be interesting to explore the relation between concerns about externalizing reactions and PTSD symptomatology in a more gender-balanced sample, as our sample had a relatively high proportion of women. A substantial proportion of patients expressed high concern for outcomes related to externalizing reactions, for instance 12% was concerned about ‘hurting someone else’ and over 25% about ‘hitting or kicking’. Addressing these concrete concerns can therefore also be of relevance in PTSD treatment.

The third factor comprised items referring to concerns about physical reactions. Patients with PTSD often experience (intense) bodily sensations, either in response to trauma-related stimuli or due to heightened physical arousal (American Psychiatric Association, 2013). A catastrophic misinterpretation of these symptoms, as is also seen in panic disorder (Austin & Richards, 2001), may lead to high concern for these negative outcomes. Panic symptoms, including panic attacks, are frequently reported by patients with PTSD (Teng et al., 2013). However, although significant, this factor showed a weak association with PTSD symptoms. Concerns about concrete physical reactions (such as dying of a heart-attack) upon exposure to trauma-reminders may especially resonate with a subgroup of patients with PTSD.

We found that threat expectancies assessed with the TAPS strongly diminished following intensified PE, in the full measure and its three subscales. Furthermore, a reduction of threat expectancies was related to a reduction of PTSD symptoms. Specifically for exposure therapy, the interest in negative threat expectancies has increased under the influence of the inhibitory learning approach to exposure therapy (Craske et al., 2008, 2014, 2022). This approach emphasizes expectancy violation as a crucial mechanism of inhibitory learning during exposure therapy. Identifying negative expectancies is thereby an important aspect. In clinical practice, patients with PTSD often find it difficult to identify concrete and testable negative outcomes they are (most) worried about. Although items in the TAPS do not necessarily refer to a biologically significant event or unconditioned stimulus (as is highlighted in the inhibitory learning approach), it may be a useful tool to initiate the conversation on threat expectancies before starting imaginal or in-vivo exercises, which can then by refined and specified to fit with the inhibitory learning approach (filling out the OptEx Nexus, see Craske et al., 2022, e.g., further concretizing what ‘unable to function’ may look like). Beyond exposure therapy, the reduction of elevated threat expectancies upon exposure to trauma reminders may represent a common underlying mechanism shared across various psychotherapeutic treatment approaches for PTSD. Administering the TAPS during other evidence-based treatments for PTSD, such as Eye Movement Desensitization and Reprocessing (EMDR) therapy or Cognitive Processing Therapy (CPT) would be valuable.

This study has several limitations and strengths. A first limitation is the relatively small size of the patient sample, which prevented us from analyzing the factor structure of the TAPS in this sample only. Second, the questionnaire was developed in the Dutch language. Third, the scale could benefit from further refinement. Our first factor (‘losing control’) contains one item that does not refer to internal threat (one’s own reactions) but rather to an external threat (‘becoming a victim again/being in danger again’). Outcomes related to external threat are underrepresented in this list, although it is an important domain of posttraumatic cognitions (e.g., ‘the world is a dangerous place’). Other future-oriented threat measures in anxiety-based disorders also seem to identify factors related to both individuals’ own reactions and external influences (Hicks et al., 2005; Scheveneels & Carpentier, 2024; Schultz et al., 2006). The addition of concerns for external threats may be clinically useful (e.g., getting physically/sexually attacked; socially rejected). In the current measure, it was difficult to add standardized expectancies related to external threat, as these depend on the type of traumatic exposure. Further research is needed to confirm the factor structure of the TAPS via confirmatory factor analysis in an independent sample. Furthermore, future work should assess whether the TAPS demonstrates incremental validity beyond a more global measure of pessimism, such as the Life Orientation Test-Revised (LOT-R; Hinz et al., 2017). A strength is that we introduce a novel measure to refine the assessment of trauma-related cognition, and show that it appears reliable, valid and relevant in the context of treatment. Additionally, the development of the items was largely data-driven, using patient responses from a large previously collected dataset, ensuring their clinical relevance. The development of this scale contributes to our understanding of negative expectancies in relation to trauma reminders in patients with PTSD.

The TAPS is a promising measure to assess trauma-related, concrete and negative expectancies, an important subcategory of posttraumatic cognitions. Outcomes showed that most patients with PTSD have multiple high concerns about negative outcomes when being confronted with trauma reminders. A three-factor solution best fitted the TAPS, where the factors ‘losing control’, ‘externalizing reactions’, and ‘physical reactions’ were identified. The TAPS, and its subscales strongly decreased following treatment and this decrease was related to a decrease in PTSD symptomatology, highlighting the relevance of the measure in a treatment context. The current findings need to be replicated, ideally in larger and more diverse patient samples. The TAPS may serve as a helpful clinical tool to identify specific threat expectancies and tailor therapeutic interventions.

## Electronic Supplementary Material

Below is the link to the electronic supplementary material.

Supplementary Material 1

## Data Availability

Due to the sensitivity of the data, the anonymized data that support the findings of this study are available from the corresponding author, MJK, upon reasonable request.
