# Supplementary material for: ‘If I am Reminded of my Trauma, I will …’: Assessing Threat Expectancies for Being Confronted with Trauma Reminders
Source: Cognit Ther Res. 2025 Feb 20;49(4):769–79. doi: 10.1007/s10608-025-10582-5 (PMC12287127; doi:10.1007/s10608-025-10582-5)
Supplement: Supplementary file 1 — Supplementary Material 1 [file 10608_2025_10582_MOESM1_ESM.docx]

**Supplement**

“**‘If I am reminded of my trauma, I will…’: Assessing threat expectancies in response to trauma reminders in PTSD patients.**”

Marike Kooistra, Agnes van Minnen, Danielle Oprel, Maartje Schoorl, Willem van der Does, and

Rianne de Kleine

**Supplement: EFA in the combined sample**

Based on eigenvalues and the scree method, we explored a two, three and four-factor structure. The three-factor structure, which we deemed to provide the best fit, is described in the main manuscript of our paper. In this supplement, we provide a summary table of the factor solutions (see Table S1) and we provide the outcomes of the two and four-factor structure.

**Table S1**

Summary of factor solutions of combined sample

| Factors | 2 | 3 | 4 |
| --- | --- | --- | --- |
| Cross-loading items | 0 | 0 | 2 |
| Non-loading items | 1 | 3 | 3 |
| Low communality items | 7 | 4 | 0 |
| Factors well-defined_._ | 2 of 2 | 3 of 3 | 3 of 4 |
| Variance explained | 44.92% | 50.38% | 52.83% |

Notes. Cross-loading items are items with factor loadings >.40 on two or more factors. Non-loading items are items that have no factor loading >.40 on any factor. Low communality are items with communality <.40. Factors that are well defined are factors with a minimum of three items.

**Table S2**

Two-factor structure in combined sample

| Items | | Factor 1 | Factor 2 | Communalities |
| --- | --- | --- | --- | --- |
| 12. | Unable to think (having a black-out) | **.885** | -.163 | **.63** |
| 24. | Unable to function | **.831** | -.067 | **.63** |
| 9. | Not knowing where I am | **.763** | -.005 | **.58** |
| 11. | Unable to talk | **.739** | -.013 | **.53** |
| 8. | Fainting | **.723** | -.131 | **.42** |
| 7. | Unable to move | **.708** | -.042 | **.46** |
| 14. | Unable to feel anything | **.700** | -.012 | **.48** |
| 17. | Collapsing | **.669** | .061 | **.50** |
| 20. | Speaking gibberish | **.603** | .095 | **.44** |
| 23. | Moving uncontrollably | **.585** | .087 | **.41** |
| 5. | Becoming a victim again/being in danger | **.582** | .024 | .36 |
| 3. | Vomiting | **.553** | -.025 | .29 |
| 15. | Hurting myself | **.537** | .196 | **.46** |
| 6. | Choking | **.536** | .149 | **.41** |
| 4. | Having a heart attack | **.529** | .046 | .31 |
| 18. | Dying | **.511** | .027 | .28 |
| 19. | Unable to stop crying | **.444** | .115 | .27 |
| 16. | Wetting or soiling my pants | **.408** | .090 | .22 |
| 21. | Walking away or running away | .377 | .284 | .36 |
| 2. | Throwing things | .031 | **.773** | **.63** |
| 1. | Screaming | -.051 | **.773** | **.55** |
| 10. | Hitting or kicking | .069 | **.753** | **.64** |
| 13. | Swearing or cursing | .017 | **.667** | **.46** |
| 22. | Hurting someone else | .130 | **.584** | **.45** |
| Eigenvalue | | 9.07 | 6.15 |  |
| (Additional) % of variance | | 39.24 | 5.68 |  |

**Table S3**

Four-factor structure in combined sample

| Items | | Factor 1 | Factor 2 | Factor 3 | Factor 4 | h^2^ |
| --- | --- | --- | --- | --- | --- | --- |
| 11. | Unable to talk | **.886** | -.003 | -.097 | -.085 | **.66** |
| 12. | Unable to think (having a blackout) | **.840** | -.101 | .000 | .134 | **.70** |
| 7. | Unable to move | **.745** | -.038 | .046 | -.105 | **.53** |
| 24. | Unable to function | **.734** | -.012 | .086 | .086 | **.65** |
| 14. | Unable to feel anything | **.715** | .017 | .009 | -.015 | **.53** |
| 9. | Not knowing where I am | **.636** | .022 | .192 | -.017 | **.59** |
| 5. | Becoming a victim again/being in danger | **.566** | .051 | .031 | .009 | **.38** |
| 20. | Speaking gibberish | **.472** | .150 | .094 | .144 | **.46** |
| 15. | Hurting myself | **.467** | .220 | .098 | .025 | **.46** |
| 23. | Moving uncontrollably | .394 | .101 | .274 | -.014 | **.42** |
| 21. | Walking away or running away | .376 | .346 | -.098 | .188 | **.44** |
| 19. | Unable to stop crying | .346 | .208 | -.068 | .336 | **.41** |
| 1. | Screaming | -.115 | **.807** | .006 | .162 | **.62** |
| 2. | Throwing things | -.016 | **.732** | .142 | -.033 | **.62** |
| 10. | Hitting or kicking | .174 | **.699** | .046 | -.217 | **.67** |
| 13. | Swearing or cursing | .102 | **.675** | -.110 | .052 | **.50** |
| 22. | Hurting someone else | .023 | **.541** | .316 | -.240 | **.56** |
| 4. | Having a heart attack | -.056 | .021 | **.821** | .009 | **.64** |
| 18. | Dying | .075 | -.010 | **.673** | -.088 | **.50** |
| 8. | Fainting | .167 | -.069 | **.547** | .350 | **.58** |
| 16. | Wetting or soiling my pants | .040 | .077 | **.528** | -.034 | **.34** |
| 6. | Choking | .251 | .137 | **.447** | -.070 | **.47** |
| 3. | Vomiting | .055 | .048 | **.444** | .369 | **.43** |
| 17. | Collapsing | .285 | .110 | **.410** | .177 | **.54** |
| Eigenvalue | | 8.30 | 5.86 | 5.74 | 1.48 |  |
| (Additional) % of variance | | 39.53 | 5.91 | 5.22 | 2.17 |  |
